# CONTEXTS.py (*CS.py*): A supervised contextual post-classification method to access multiple dimensions of complex geospatial objects

**DOI:** 10.1016/j.mex.2024.102753

**Published:** 2024-05-16

**Authors:** Vincenza Ferrara, Johan Lindberg, Anders Wästfelt

**Affiliations:** a Department of Archaeology and Ancient History, Uppsala University - Engelska Parken, Thunbergsvägen 3H, Uppsala 751 26, Sweden; b Department of Human Geography, Stockholm University - Geovetenskapens hus, Svante Arrhenius väg 8, Frescati, Stockholm 106 91, Sweden; c Novilla 1, Bålsta 746 32, Sweden

**Keywords:** Conceptual spaces, Heterogeneous landscape, Land use, Land cover, CONTEXTS.py (*CS.py*) - A supervised contextual post-classification method

## Abstract

The qualitative dimensions of visible features in space can be captured by connecting spatial configurations arranged in a variety of different ways to diverse *conceptual spaces*. By conceptual spaces, we intend mental concepts describing specific spatial configurations present in a geographical area, defined by the contextual relationships among their constitutive elements.

This paper presents a new supervised post-classification method allowing the extraction of semantically complex spatial objects from a single image of the Earth as, for instance, diverse conceptual spaces referring to multiple dimensions of land use (temporal, cultural, social, etc.).

Computationally, our method is operationalised by CONTEXTS.py (*CS.py*), a plugin written in Python for QGIS. *CS.py* relies on training areas, defined by the user at diverse scales, to identify and extract in the input image conceptual spaces whose spatial contexts have the same spatial features present in the training areas.

Applied to a case study on the island of Sicily, where millennial land use dynamics have resulted in a mosaic landscape, *CS.py* could detect from an orthophoto diverse conceptual spaces of land use in an area ordinarily classified as one land cover, thus expanding the capabilities of geospatial analysis to reach additional qualitative dimensions of information from image data.

• *CS.py* simplifies a supervised contextual post-classification routine in an easy-to-use, practical and accessible QGIS plugin;
• *CS.py* joins a family of tools for supervised object-based classification (e.g. OTB, GRASS), providing, additionally, the possibility to include contextual information as spatial criteria to train the classification routine.
• *CS.py* has broad applications in different disciplines investigating landscape from quantitative and qualitative perspectives, allowing both, as in multiple environments.

## Graphical abstract

**Figure:**
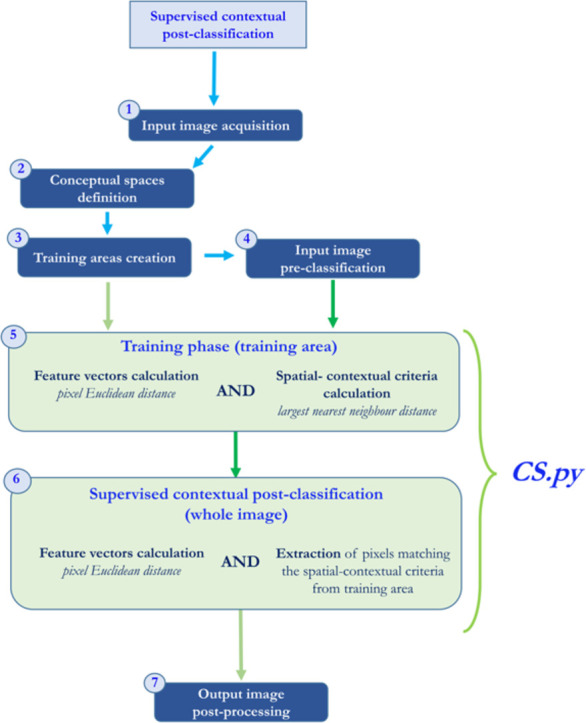

Specifications table

**Table:** 

Subject area:	Environmental Science
More specific subject area:	Remote sensing and classification
Name of your method:	CONTEXTS.py (*CS.py*) - A supervised contextual post-classification method
Name and reference of original method:	Wästfelt, A. (2009). Land use qualities identified in remotely-sensed images. International Journal of Remote Sensing, 30:9, 2411–2427. 10.1080/01431160802552694 Ahlqvist, O., Wästfelt, A., Nielsen, M. (2012). Formalized interpretation of compound land use objects – Mapping historical summer farms from a single satellite image. Journal of Land Use Science, 7(1), 89–107. 10.1080/1747423X.2010.537787 Wästfelt, A., Arnberg, W. (2013). Local spatial context measurements used to explore the relationship between land cover and land use functions, International Journal of Applied Earth Observation and Geoinformation, 23: 234–244. 10.1016/j.jag.2012.09.006
Resource availability:	*CS.py* is available from: 10.5281/zenodo.10615018 License: CC BY-NC 4.0

## Background

Whatever landscape today is the heterogeneous legacy of human-nature interactions along different temporal and spatial scales, in always-changing environmental conditions. While methods to quantify landscape heterogeneity frequently build upon the concept of spatial entropy, used to measure diversity even at multiple scales [1], entropy remains a measure of spatial heterogeneity, not an attempt to unpack and qualify such complexity. In our research, we approach landscape complexity from a qualitative perspective instead, working with the intangible layers of meanings associated with visible features in space, and we do so by connecting spatial configurations arranged in a variety of different ways to diverse conceptual spaces [2,3]. By conceptual spaces, we mean mental concepts that describe specific spatial configurations present in a geographical area, defined by the contextual relationships among their constitutive elements. Therefore, for instance, the conceptual space “intensive orchard of crop x” is defined by the specific spatial configurations of its constitutive elements and their contextual relationships. An intensive orchard could thus be characterised by trees planted in straight lines and very close to each other to quicken the harvest, usually planted on heavily tilled soil, with absence of other vegetation features and/or spatial elements. In this paper, we will demonstrate how, by operationalizing the concept of geospatial context from a computational point of view, we can access from a single image of the Earth diverse conceptual spaces referring to multiple dimensions of land use (temporal, cultural, social, etc.), even in the case of the same land cover.

Within object-based analysis (for a review cf. [[4], [5], [6]]), research goes mainly towards a refinement of segmentation and classification techniques able to reach accurate extractions of individual objects (e.g. a tree, a car, a building), without assembling these real-life different objects into computational segmented objects with higher semantic meaning (e.g. a tree with many trees — > an orchard; a tree with other vegetation features — > *a* garden; a building with roads and other buildings — > *a* neighbourhood). While the theorization and operationalization of geographical context have reached outstanding quantitative results [7], the development of classification approaches for the extraction of complex geographic objects has been so far very limited [3,[8], [9], [10], [11], [12], [13], [14]]. To fill this gap, we present CONTEXTS.py (*CS.py*), a new supervised post-classification method allowing the extraction of semantically complex spatial objects as, for instance, different conceptual spaces of the same land cover. Our method builds on previous work on spatial relational post-classification [3,8,9] that has proved the possibility to map different social-spatial distributions of land uses from the same land cover, by using the spatial context of pixels [15] as an integrative source of information together with texture (spectral reflectance) to improve final classification results [16].

## Method details

In recent work [17], we have shown how unsupervised contextual and spatial-relational analysis can be applied to extract from a single image of the Earth useful classes carrying information that, integrated with other sources of evidence (e.g. local ecological knowledge, ground truth), allows reading the multiple layers of meanings in the historical, cultural and ecological formation processes of a heterogeneous landscape. The method we present here is the supervised version of the same geospatial approach. In our method, a conceptual space such as, for instance, “intensive orchard of crop x” is categorised in terms of *context*, meaning with this word the peculiar spatial configurations of a conceptual space as defined by the contextual relationships among its constitutive elements in terms of biophysical features, human-nature processes, intentionalities and ecological dynamics. In our method, these contexts are then computed as *training areas*, whose information will be then used to set the clustering conditions of a supervised classification of the entire image data.

From a computational point of view, our method is operationalised by CONTEXTS.py (*CS.py*), a plugin for the Open-Source GIS (Geographical Information System) program QGIS, written in Python programming language.

**Fig. 1:**
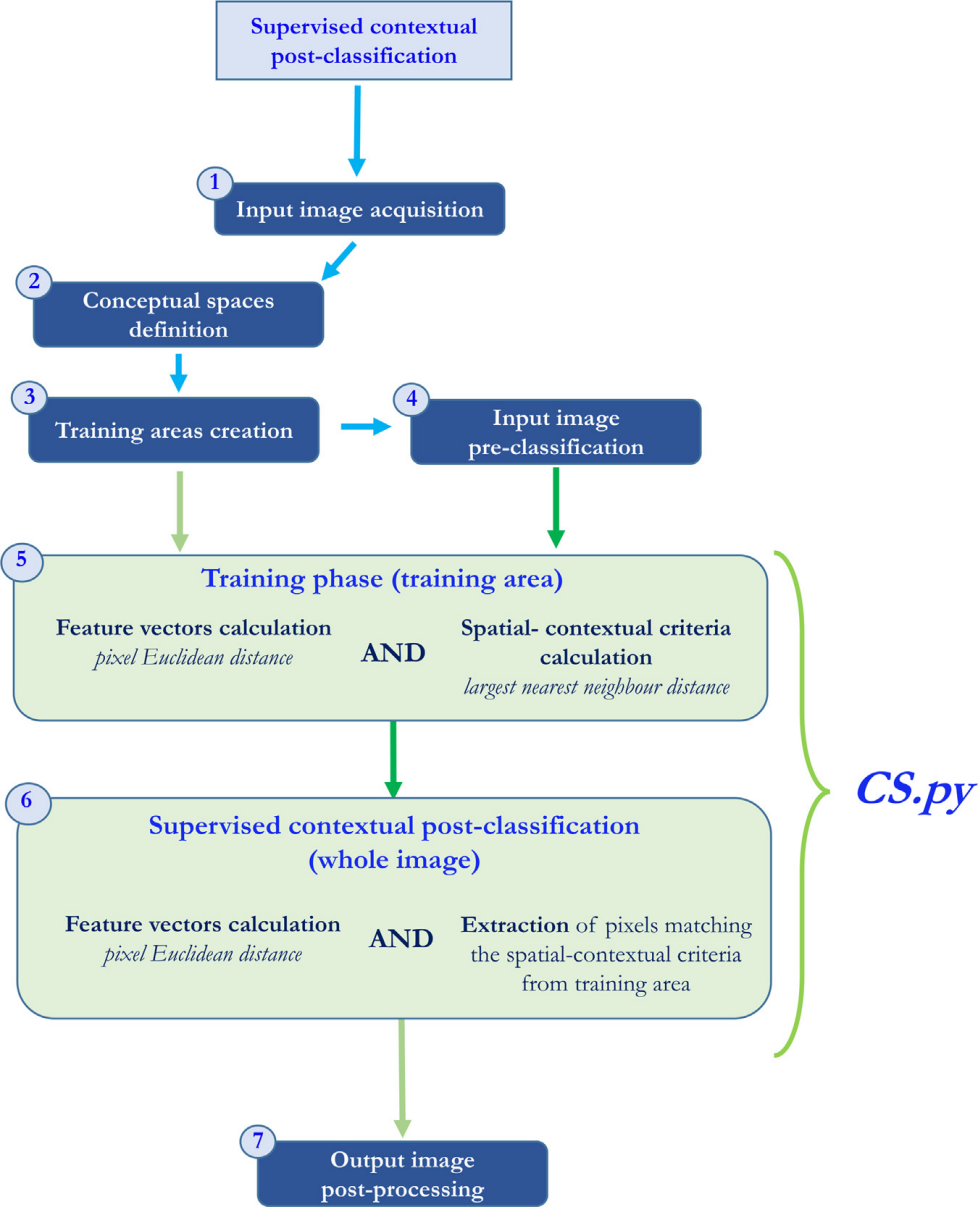
The overall supervised contextual post-classification workflow, with indication of the preliminary and post-processing steps (blue rectangles) and the analytical steps automated by *CS.py* (green rectangles).

With *CS.py,* it is possible to use training areas, created by the user at diverse and multiple scales, to identify and extract in the image data conceptual spaces whose spatial contexts have the same spatial features present in the training areas. The overall supervised contextual post-classification procedure automated by *CS.py* consists of two analytical steps: 1) Training phase and 2) Contextual post-classification, detailed below together with a description of the four preliminary steps needed to prepare the input image and training areas for the analysis, as well as post-processing stage (Fig. 1):

Step 1 (Preliminary): Input image acquisition
Step 2 (Preliminary): Conceptual spaces definition
Step 3 (Preliminary): Training areas creation
Step 4 (Preliminary): Input image pre-classification
Step 5 (Analytical): Training phase
Step 6 (Analytical): Contextual post-classification
Step 7: Output image post-processing

### Steps 1–4 (Preliminary)

The first four steps (1–4) in the supervised contextual post-classification workflow are preliminary since they are meant to prepare the input data to use in the following analytical steps automated by *CS.py*.

#### Step 1 - Input image acquisition

A georeferenced raster shall be used as input image.

**Fig. 2:**
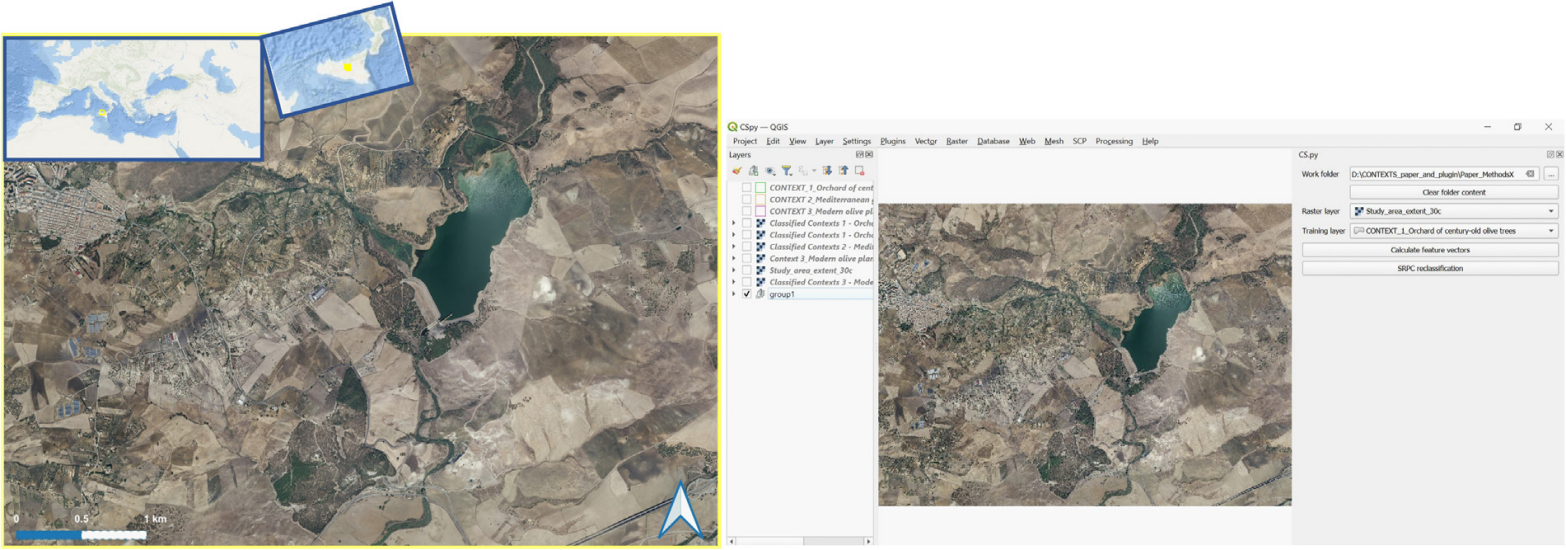
Step 1 – Input image acquisition. RGB orthophoto of the study area, year 2013, 25 cm pixel resolution, scale 1:10.000, used as input in *CS.py* (Image source: Regione Siciliana, Assessorato Regionale Territorio e Ambiente, Dipartimento di Urbanistica, Area 2 Interdipartimentale, “Elemento di proprieta’ della Regione Siciliana ceduto in data 31/01/2023 al n. 2023-S-3466”).

In the case study presented here, we used an RGB orthophoto, year 2013, 25 cm pixel resolution, scale 1:10.000 (Fig. 2). The study area, for a total coverage of ∼ 21 km^2^s, represents a landscape where land use dynamics spanning over millennia have resulted in remnants of century-old olive trees, historically located in areas where it was extremely hard or impossible to plough the land to grow cereals, and still present in a landscape today dominated by intense cereal cultivation and a social context characterised by severe depopulation [18,19].

#### Step 2 - Conceptual spaces definition

As second step in our method, the user is supposed to define the intended conceptual space(s) they would like to extract from the input image. Conceptual spaces can have diverse levels of semantic complexity (e.g. a wood, a parking lot, a farm, a commercial area and so on). It is important that the users define a conceptual space in terms of the peculiar spatial configurations of its constitutive elements and of the contextual relationships among these elements and among these elements and the elements external to the conceptual space but present in the rest of the input image. Thus, for instance, a forest can be conceptually defined as “a cluster of trees with a very high concentration of arboreal biomass, surrounded by grassland and/or bare soil and/or water, buildings, etc.”, a cereal or fallow farm may be conceptualised as “medium-large size extension of heavy tilled land (or land covered with crops, depending on the time of the year when the input image has been taken), surrounded by forest and/or lakes etc.”, and so on, depending on the users’ preferences and research scope.

The basic theoretical assumption of our supervised contextual post-classification method is that, in creating conceptual spaces, the user shall adopt the reasoning of human observers when they try to interpret and identify a complex of heterogeneous objects in an image. As pointed out by Ahlqvist et al. [3], such human cognitive process combines mentally constructed objects such as, for instance, houses, trees, different types of grass areas, and associates them to general concepts of settlement types, inferring specific forms of land use and/or spatial functions. These are what we mean by conceptual spaces, elicited in the human mind by observing patterns and clusters of spatially distributed spectral energy (deriving from the pixels of the image data). It is thus preferable that the users have some degree of previous qualitative knowledge of the area represented by the image data they intend to work with, as well as a preliminary idea of the conceptual spaces they would like to extract from the input image.

**Fig. 3:**
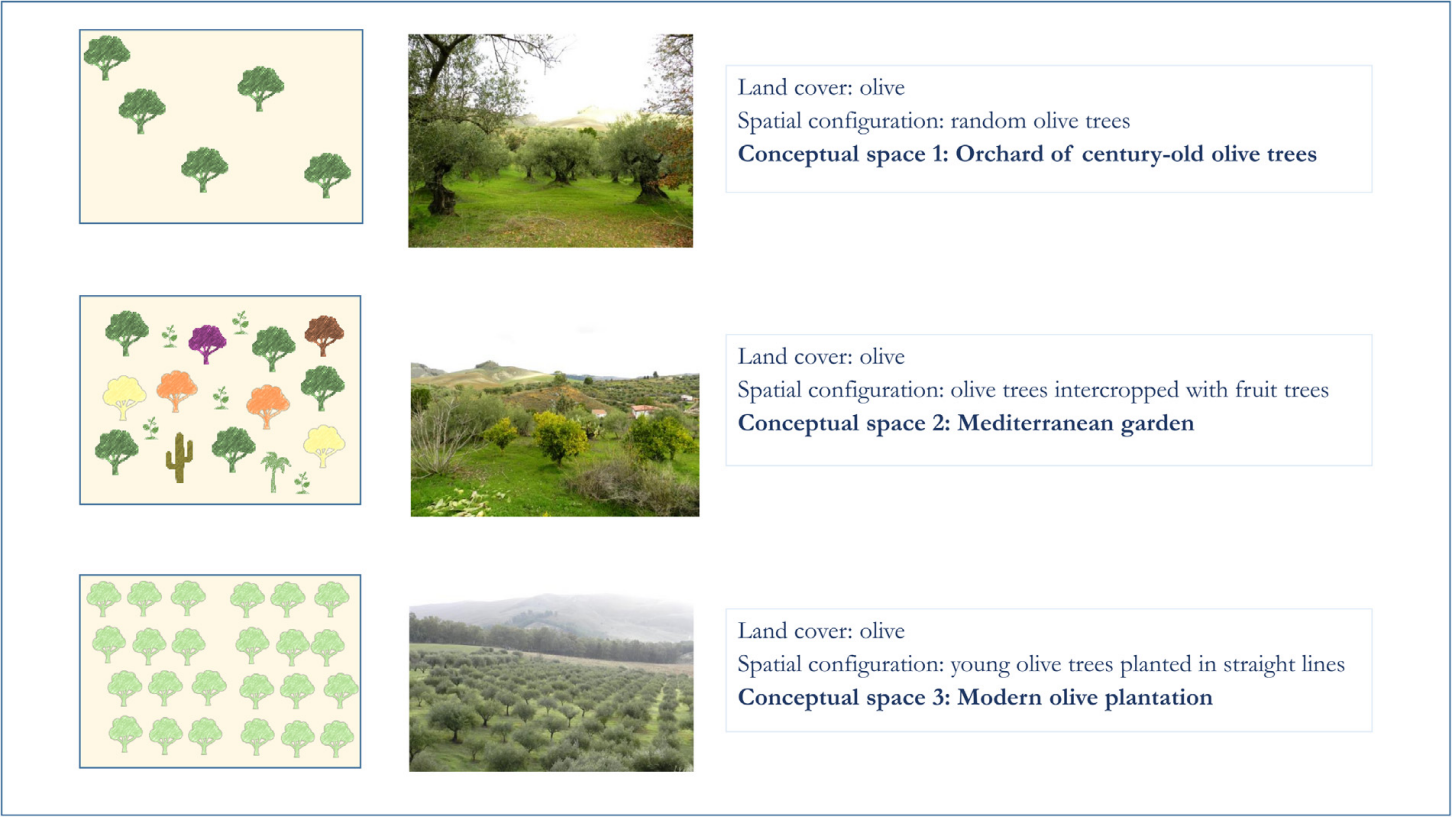
Step 2 – Definition of conceptual spaces. The image shows the three conceptual spaces centred on the local use of the olive tree that we defined for the analysis in our study area.

In the case study presented here, to test the possibility to extract different conceptual spaces of the same land use, we defined the following three conceptual spaces centred on the local use of the olive tree (Fig. 3):

1. Orchard of century-old olive trees
2. Mediterranean garden
3. Modern olive plantation

These conceptual spaces represent three different types of olive cultivation, related to different land uses, management practices and historical periods. The orchard of century-old olive trees (conceptual space 1) is a remnant of the typical past agroforestry system, where olive trees were intercropped with legumes, vegetables, other fruit trees (e.g. pear, pomegranate, almond, prunus, citrus trees) and/or used seasonally for grazing, while today it is characterised by empty spaces in between the olive trees and thus successional vegetation dynamics if the soil is not tilled.

For Mediterranean garden (conceptual space 2), we intend both century-old and younger olive trees intercropped in very small plots mainly with young citrus and almond trees. They usually are located in the garden of country houses.

An intensive modern olive plantation (conceptual space 3) is characterised by the presence of a high density of young olive trees planted in rows and close to each other, usually on plain terrains, so to facilitate the harvest. Usually, these plantations are characterised also by the absence or very low presence of other vegetation elements (e.g. fruit trees).

#### Step 3 - Training area creation

**Fig. 4:**
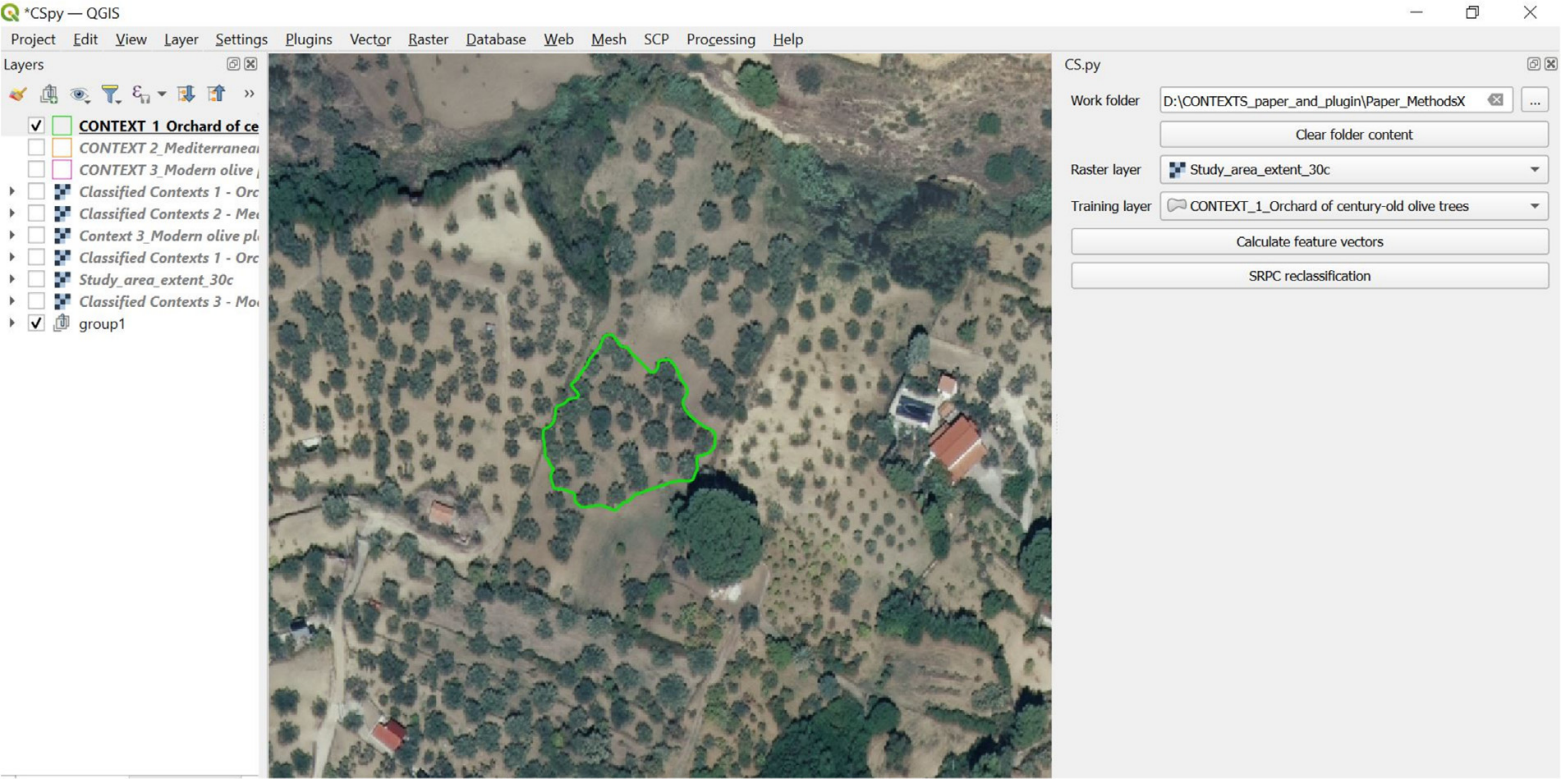
Step 3 - Training area creation to delimit the intended conceptual space defined by the user. The image shows the training area representing the conceptual space “Orchard of century-old olive trees”.

Once defined, a conceptual space is computationally created by the user as a *training area* in *CS.py*. In simple words, the user creates a polygon (in QGIS shapefile format) to delimit on the input image the defined conceptual space. This polygon will be used as a training area in the following analytical steps (steps 5 and 6) of the supervised post-classification routine. Fig. 4 shows the example of the training area corresponding to the conceptual space “Orchard of century-old olive trees”, defined by the authors. In Table 1 are shown all the three training areas created by the authors, corresponding to the three conceptual spaces (“Orchard of century-old olive trees ”,“ Mediterranean garden” and “Modern olive plantation”) defined in terms of their contextual elements.

**Table 1:** The three conceptual spaces (“Orchard of century-old olive trees”, “Mediterranean garden” and “Modern olive plantation”) defined in terms of their contextual elements and relative training areas created to use as input data for the analytical steps in *CS.py*.

Conceptual spaces	Contexts	Training areas
**1. Orchard of century-old olive trees**	Orchard of big century-old olive trees, lying scattered within small plots (∼ 0.5 or 1 hectare). Presence of few other elements (fruit trees). Soil in between the trees can be tilled, but in some cases, overgrowth vegetation is present (sign of the abandonment process).	
**2. Mediterranean garden**	Tree polyculture made of century-old and young olive trees, mainly citrus and almond trees, other fruit and garden trees. Very small distance among the trees. Soil is tilled and not covered by vegetation. Usually, this polyculture is close to a country house.	
**3. Modern olive plantation**	Modern olive orchards made of young trees, usually planted in straight rows. Very low presence or absence of century-old olive trees and/or other vegetation elements. If old olive trees are present, they are randomly distributed in space, and usually, the new trees are planted in line with them, if possible. Soil is usually heavily tilled in between the trees, absence of cover vegetation.	

#### Step 4 - Input image pre-classification

Step 4 entails a pre-classification of the input image the user intends to work with. This step is performed for two reasons. The first aim is to take on board, in the following analytical steps, also the spectral information provided by the input image while reducing, at the same time, the amount of input data into a manageable size. Secondly, a pre-classification of any sort appears to be a necessary procedure because the next step in the workflow is represented by the calculation of distances between different image objects (and their pixels). In the case of the supervised contextual post-classification method presented here, the image objects consist of spectral classes and distances are calculated among pixels belonging to different classes. Thus, a preliminary spectral classification is a suitable preparation step.

Previous works done with the same method (cf. [3]), as well as with an unsupervised version of it [17,20] have shown it is preferable to use an unsupervised classification algorithm to prepare the input image for further steps.

**Fig. 5:**
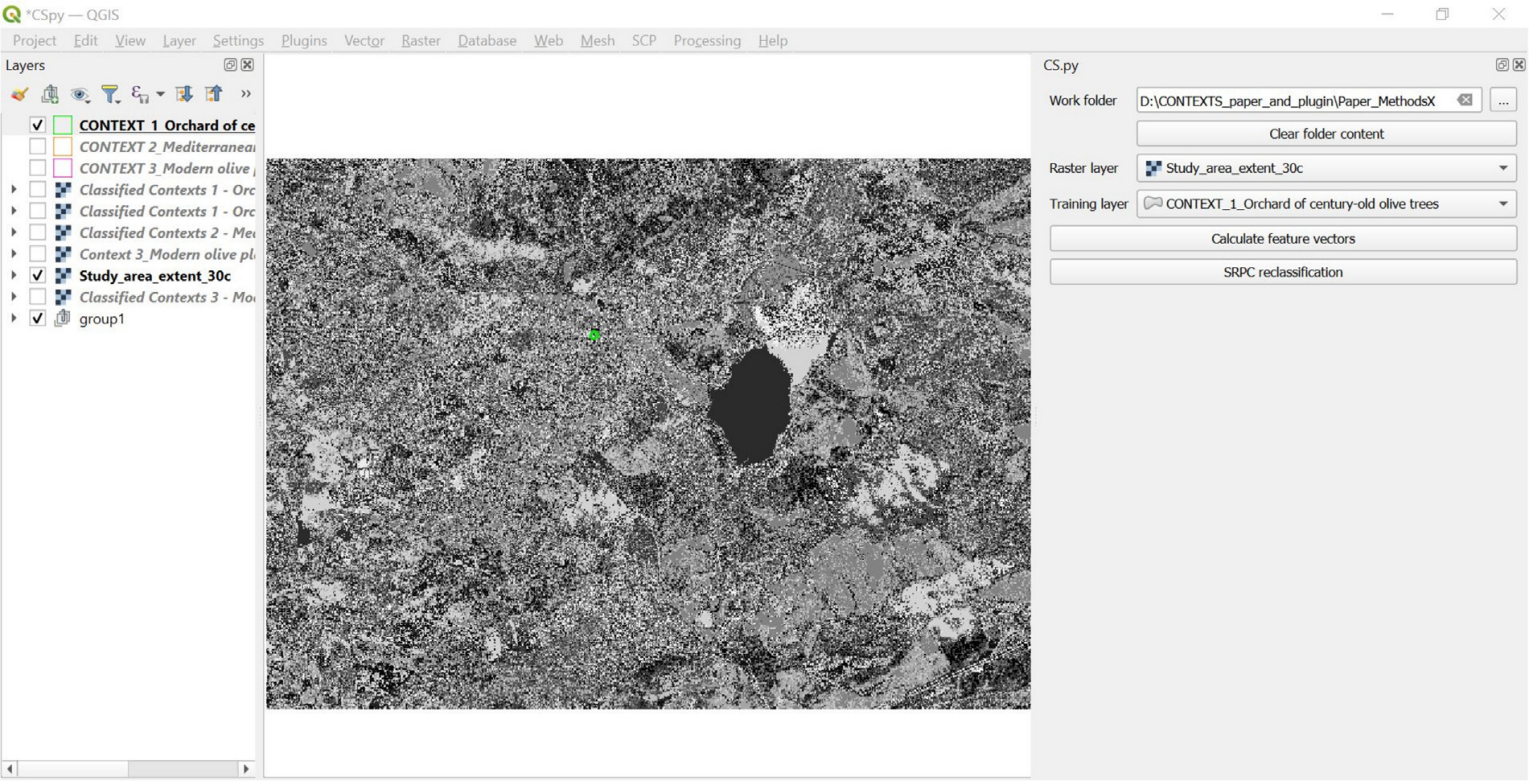
Step 4 – Input image pre-classification. In this example, the input image was pre-classified into 30 classes with the K-means algorithm.

In the case study presented here, the orthophoto used as input image has been reduced into thirty (30) spectral classes with an unsupervised KMeans classification from Orfeo ToolBox (Fig. 5).

There is no limit on the amount of classes that the input image could be classified into, but it shall be taken into account that the larger the number of classes in the input image the heavier the computational processing done during the *CS.py* steps, as well as the storage need. From earlier studies [8], we also know that including a large number of classes does not necessarily improve the thematic precision of the results given by the supervised contextual post-classification routine.

### Step 5 and step 6 (Analytical)

#### Step 5 – training phase. feature vectors and spatial-contextual criteria calculation in the training portion of the image

**Fig. 6:**
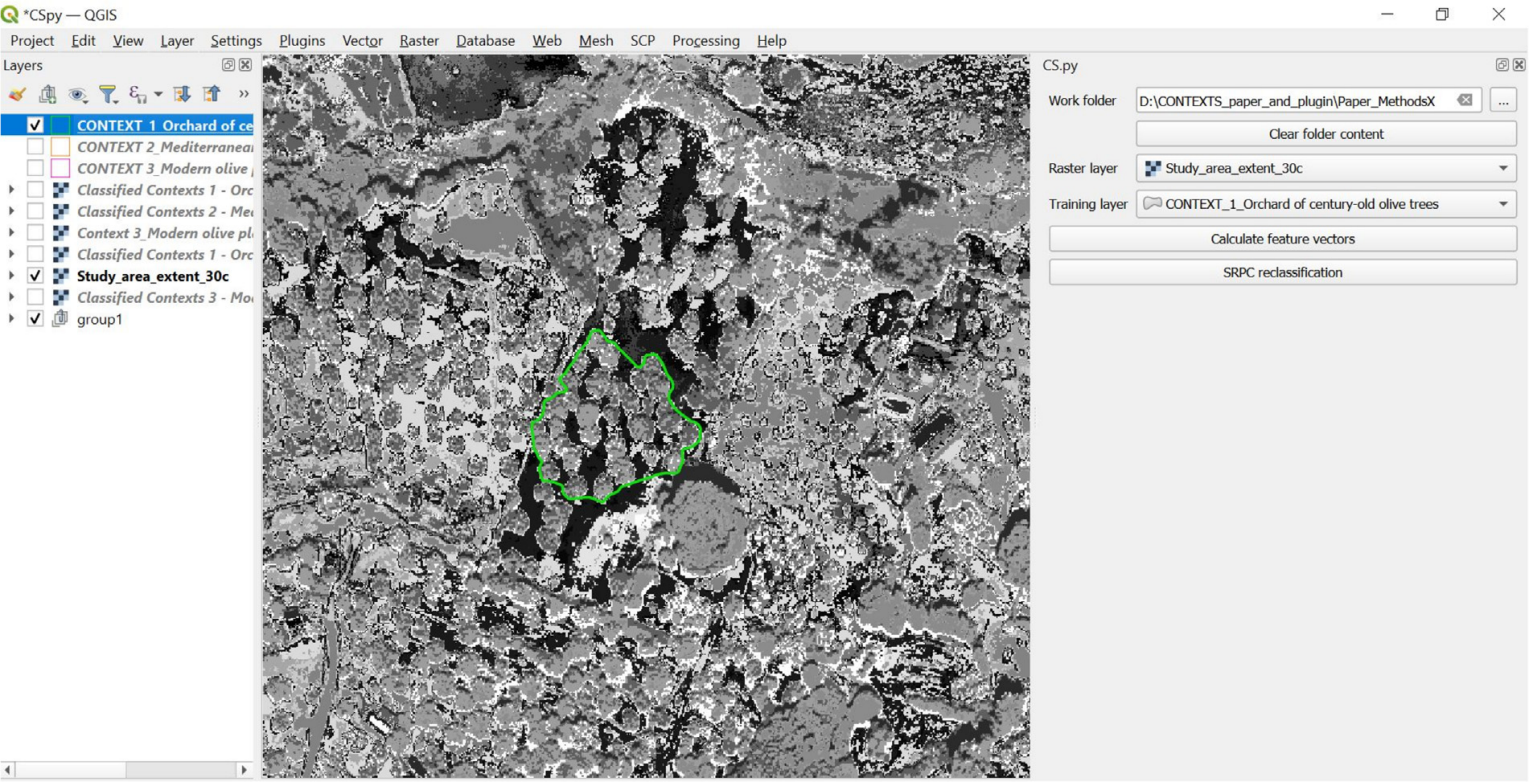
Example of portion of the input image corresponding to training area created in Step 3 (in this case, the training area represents conceptual space 1, “Orchard of century-old olive trees”).

In this step, the portion of the input pre-classified image corresponding to the training area (created in Step 3) is taken into consideration (Fig. 6). The aim of Step 5 is to define the training area criteria to use for the contextual post-classification of the whole input image (which will be done in Step 6).

**Fig. 7:**
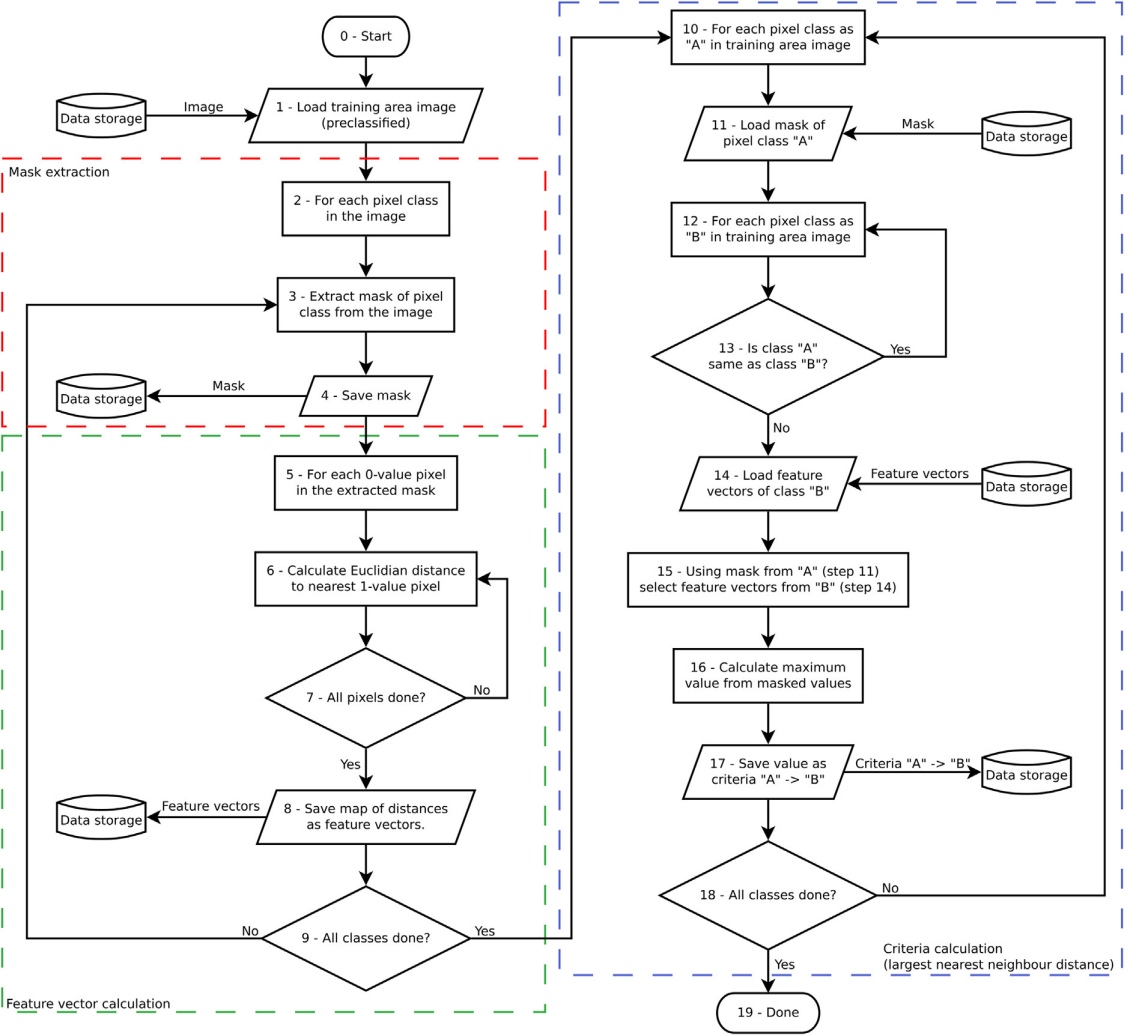
Flowchart of the feature vector and the spatial-relational criteria (the largest nearest neighbour distance) calculation done by *CS.py* in the training area of the image.

For each of the pixels of this pre-classified image, the so-called “contextual feature vectors” are calculated (cf. feature vector calculation in flowchart, Fig. 7). The contextual feature vector is meant to contain information about the pixel´s surroundings. Specifically, the contextual feature vector contains information about each pixel´s geometric (Euclidean) distance (nearest-neighbour distance) from each pixel to all the nearest pixels of all the different classes derived from the unsupervised pre-classification of the input image (done in Step 4).

In our methodological approach, calculating the nearest-neighbour distance is a computational way to describe the contextual surroundings for each pixel. In the case of a pre-classified image in 30 classes, that means a 30-dimensional contextual feature vector per each pixel, with each dimension constituting the nearest distance of the pixel to each of the classes, and together all these dimensions make up the contextual surroundings of the pixel. This means that each and every pixel in the image has a location, in the feature space, which is created by these thirty dimensions. Such dimensions will be further analysed and processed in the next step of the supervised contextual post-classification routine.

**Fig. 8:**
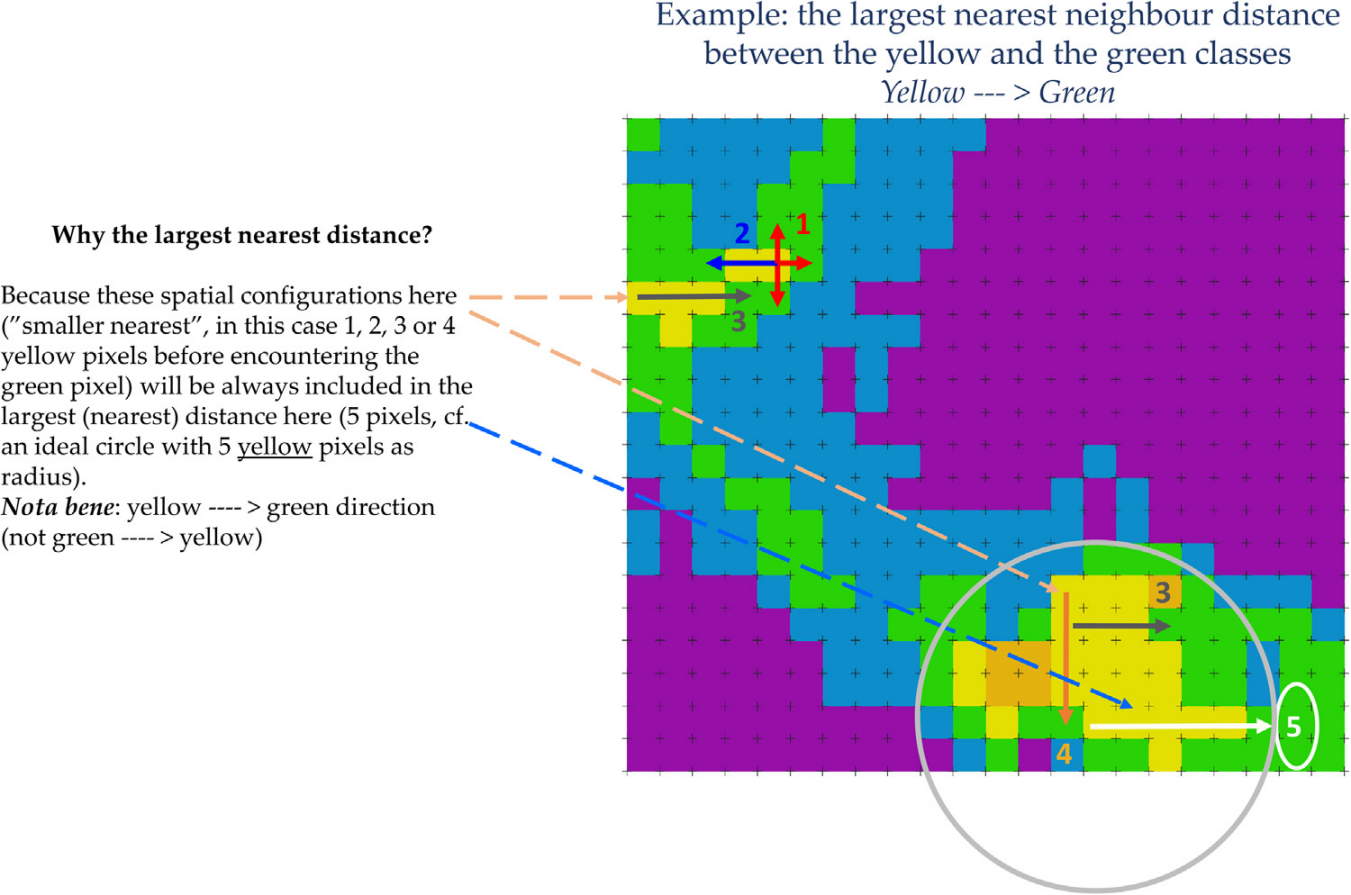
Exemplification of the concept of the largest nearest neighbour distance [8] between pixels belonging to two different classes (yellow and green classes) within a hypothetical training area.

It follows now a training phase, in which *CS.py* calculates the spatial-contextual criteria from the training area (cf. spatial criteria calculation in flowchart, Fig. 7). The contextual information used as a spatial criterion is the *largest nearest neighbour distance* [8]. The largest nearest neighbour distance (or the smallest nearest distance able to cover the largest area) is calculated by first calculating all nearest neighbour values between all the pixels of the classes in the training area, and secondly by keeping only the largest for each neighbour relation (Fig. 8).

**Fig. 9:**
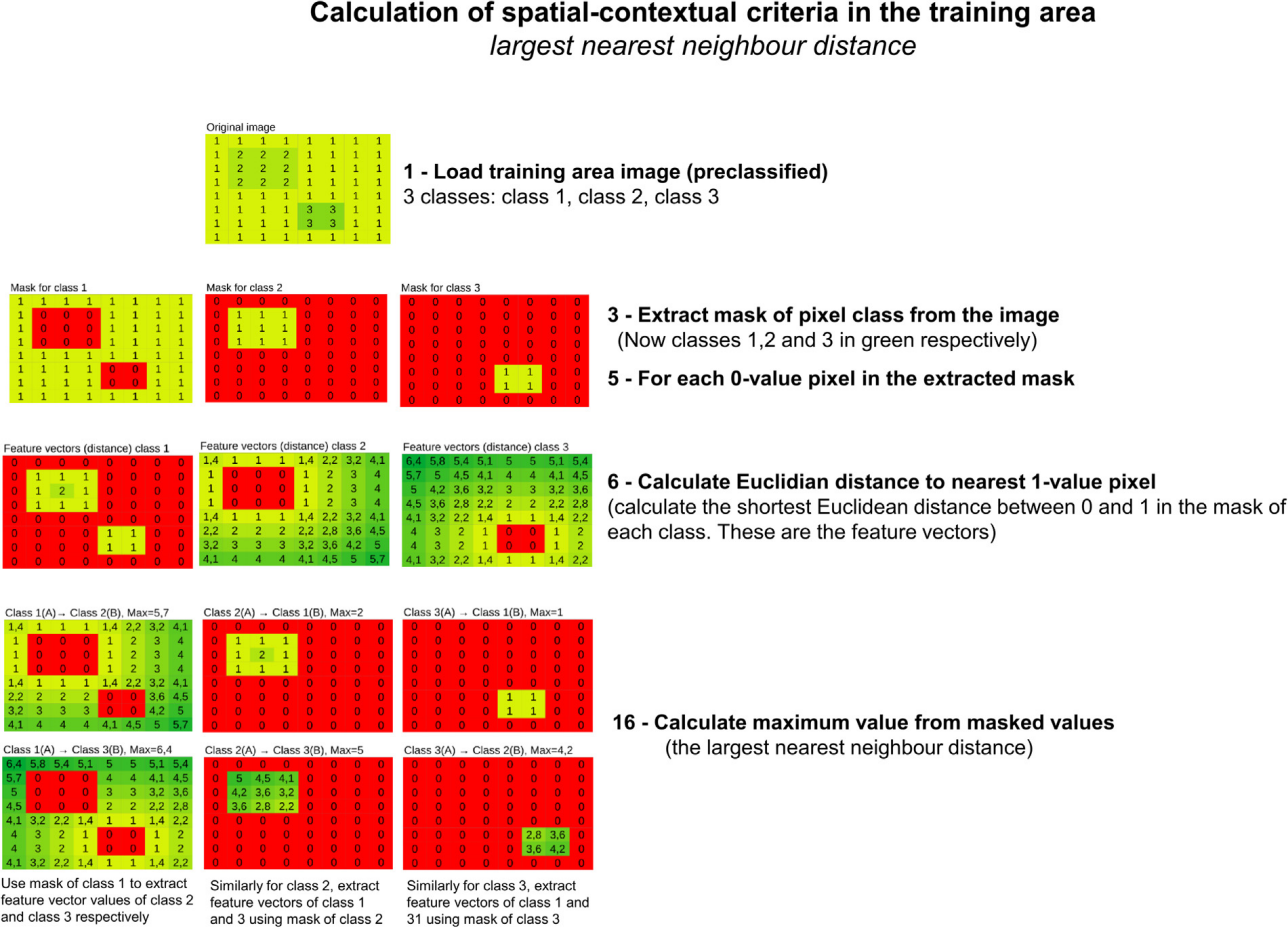
Exemplication of the spatial-contextual criteria calculation done by *CS.py* (cf. flowchart Fig. 7) for all the pixels of a training image ideally classified into three different classes (class 1, class 2 and class 3).

A simplified representation of the computational procedure for the calculation of the spatial-relational criteria is provided in Fig. 9.

#### Step 6 – supervised contextual post-classification

**Fig. 10:**
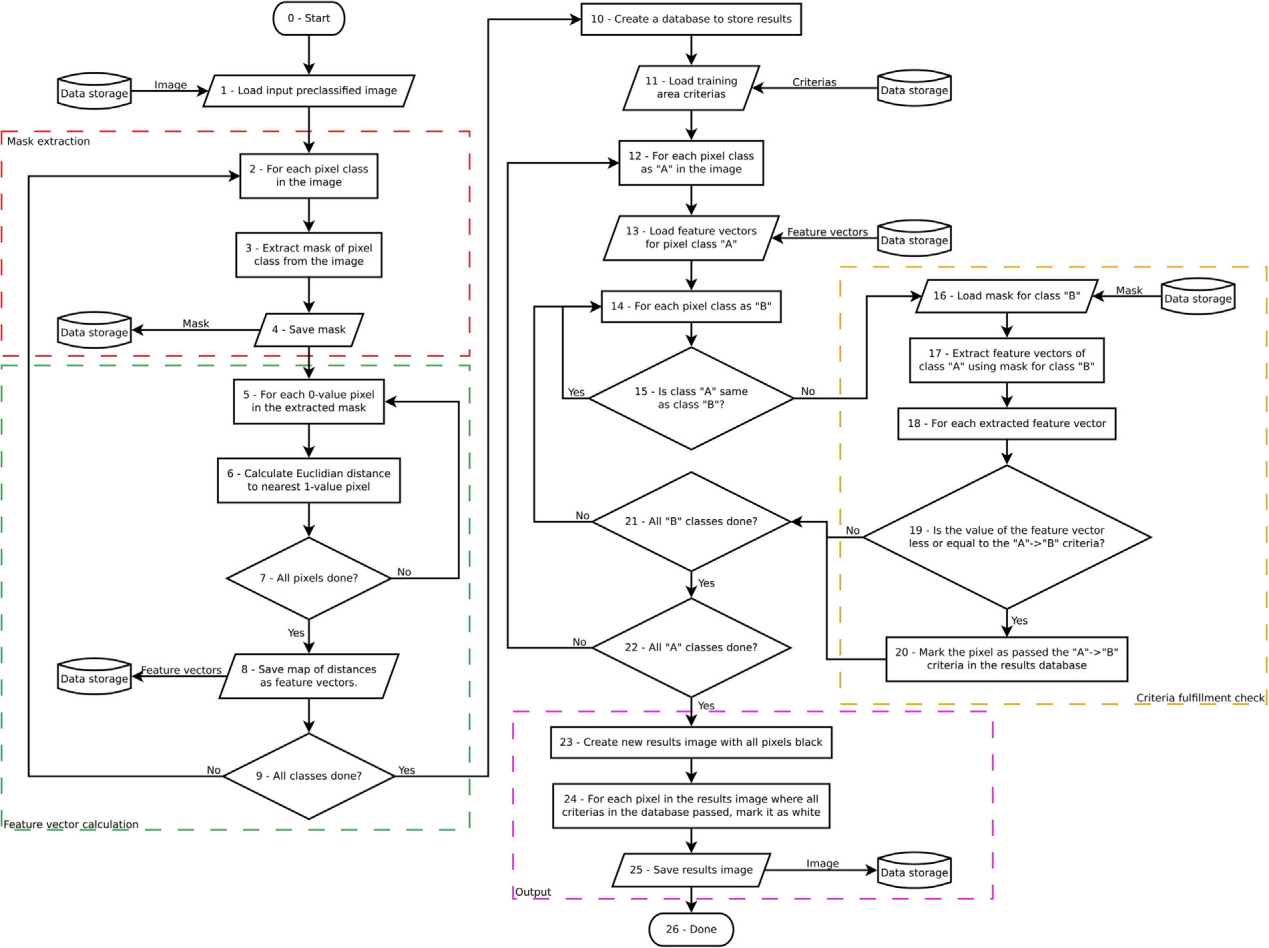
Flowchart of the supervised contextual post-classification step workflow done by CS.by.

Step 6 represents the post-classification phase,in which *CS.py* uses the spatial-contextual criteria calculated in the training phase (Step 5) to re-classify each and every pixel in the input image according to these criteria and their contextual feature vectors (cf. flowchart in Fig. 10).

After the calculation of the feature vectors for the entire image as in Step 5, now a reclassification of the input image is done pixel by pixel. Every pixel that has a spectral class represented in the training area is evaluated based on its nearest neighbour (Euclidean) distance to different spectral classes (information stored in the contextual feature vectors) and the largest nearest neighbour distances calculated for each pixel in the training area.

If a pixel outside of the training area has the same spectral values of a pixel inside the training area, but has a nearest neighbour distance that is larger than the largest nearest neighbour distance found in the training areas for that spectral class, it is classified as having a different spatial context compared to the pixels of the training area. In other words, although this pixel has the same spectral class as a pixel in the training area, it is here considered as representing a different feature because of its different spatial context (its nearest neighbour distance is larger than the largest nearest neighbour distance found in the training areas for that spectral class). Only pixels having a spatial context that corresponds to the spatial context of the pixels in the training area are considered as signalling the presence of on-the-ground features similar to those embodied in the training area. In such a way, using these simple spatial-contextual criteria, it is thus possible to separate pixels from the same class into two or more target classes that have similar spectral and spatial context of the training area.

**Fig. 11:**
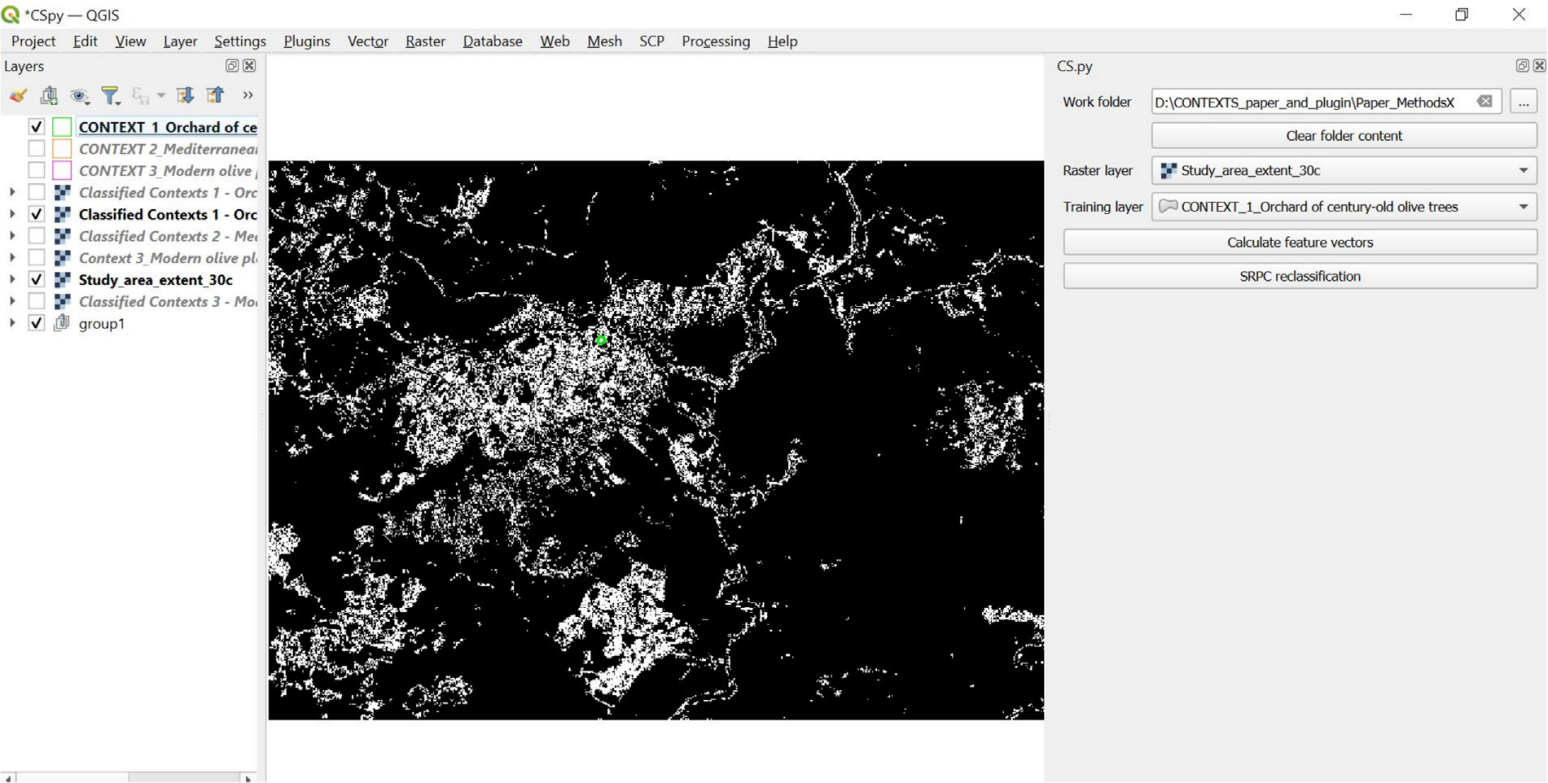
Output raster image given by *CS.py* after the post-classification done on the input pre-classified image, in accordance with the spatial-contextual criteria extracted by the training area. In this example, the training area represents conceptual space 1, “Orchard of century-old olive trees”. This output raster image, as result of the post-classification, indicates with class 1 (white) all the pixels that match the spatial-contextual criteria defined by the training area (in simple words, all the pixels that have the same spatial context as the ones present in the training area, orchards of century-old olive trees).

The result of this post-classification will be a new image (Fig. 11), in which every pixel is assigned a new class (“1” or “0”), where 1 means that the pixel has the same spatial context of the pixel in the training area used to run the post-classification. In Fig. 11, these pixels are represented in white.

In other words, only the pixels from the contextual feature vectors that fulfil the set of spatial criteria defined as contextual by the training areas are extracted and classified as 1. All other pixels were coded 0 (black in the output raster image given by *CS.py*).

The users can perform Steps 5 and 6 for as many different training areas (conceptual spaces) as they intend to create at various scales for their research purposes. In our case study, we used three different training areas.

#### Step 7 – output image post-processing

**Fig. 12:**
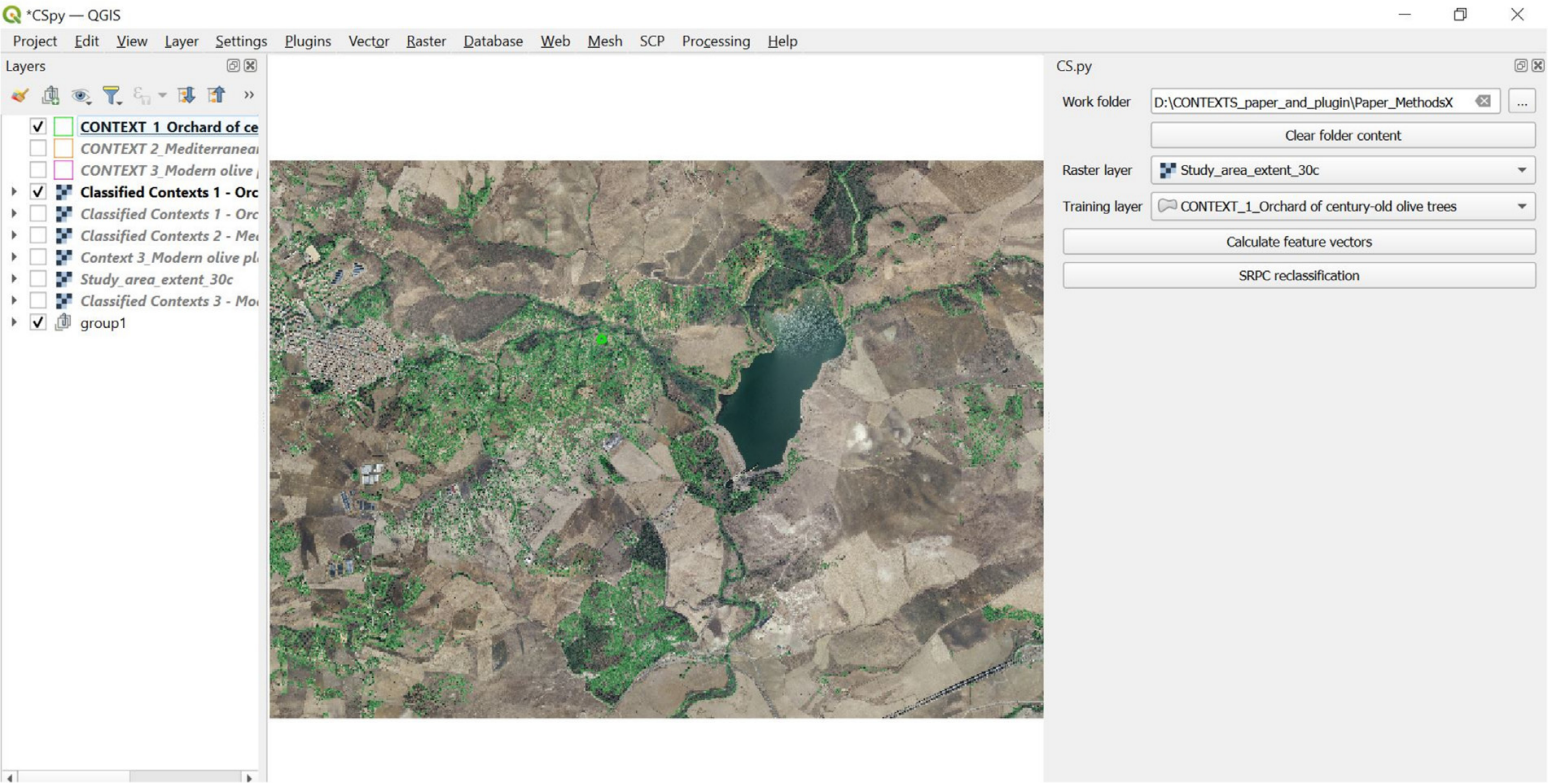
Example of post-processing of the binary image given as output by *CS.py* in Step 6.

As the output of *CS.py* is a binary raster image with only two classes (0 and 1), the users may want to post-process it in various ways, according to their research scope. Fig. 12 shows a post-processing of the output image given by *CS.py* (Fig. 11), which was vectorised and modified in the symbology for further analysis.

**Fig. 13:**
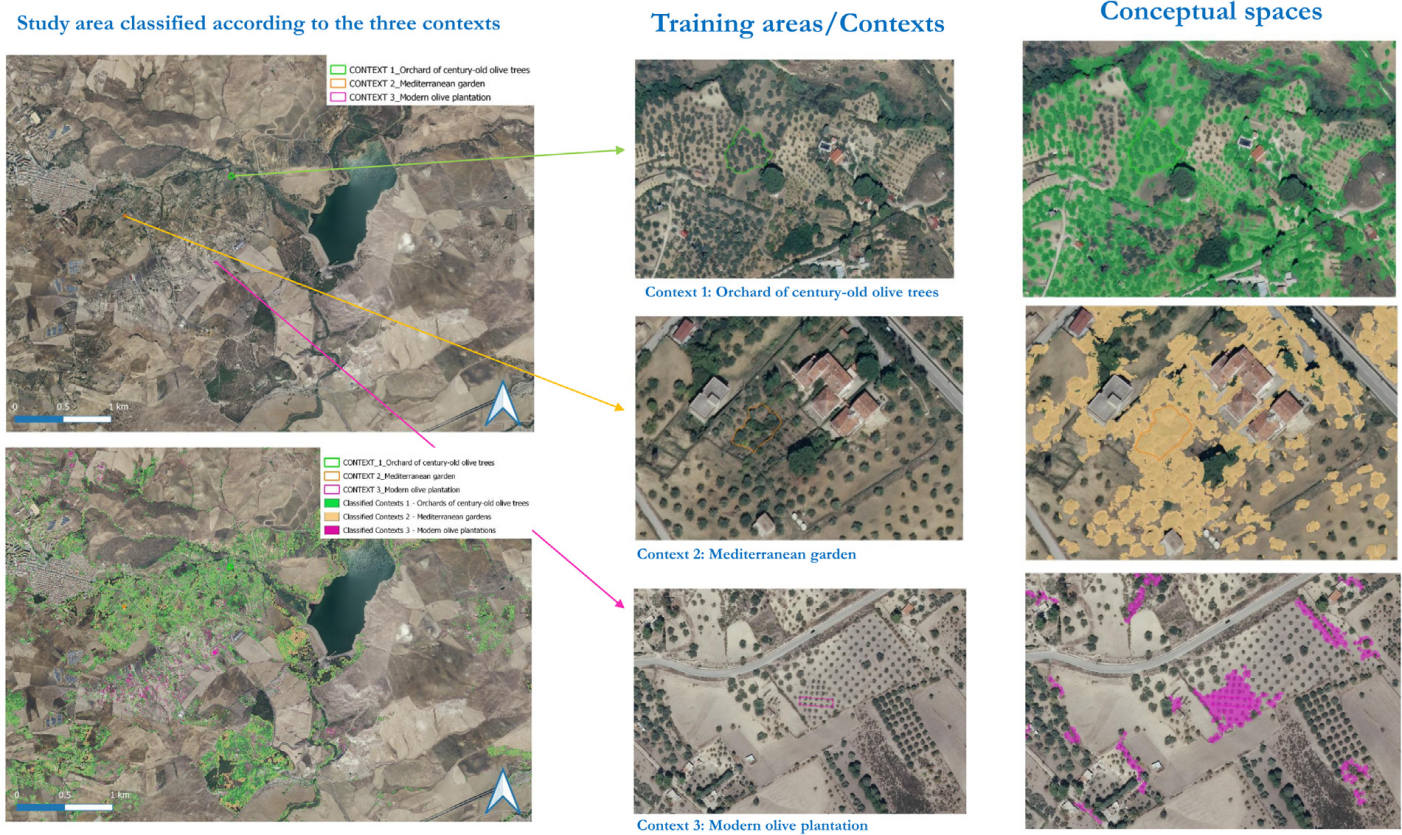
Results from the supervised contextual post-classification done with the *CS.py* tool of the input image representing the study area. From the left side, respectively: the overall study area classified according to the three training areas/contexts created, the three training areas/contexts, and the local conceptual spaces extracted according to each of the three training areas/contexts.

Furthermore, in Fig. 13 are shown the results of the post-classification done with *CS.py* on the input pre-classified image of the study area, according to the three conceptual spaces created. Per each training area/geospatial context created, Fig. 13 displays also the corresponding local conceptual spaces extracted by *CS.py* from the pre-classified input image.

Given three different contexts, all characterised by the presence of the same tree species (*Olea Europaea* L.), the results from the post-classification of the input image done with *CS.py* demonstrate that it is possible to distinguish among different conceptual spaces of the same land use (“olive groves”). The three conceptual spaces extracted from the image represent different arrangements of space, according to local contextual features and responding to diverse spatial functions, intentionalities and land management strategies, which moreover change over time (with Context 1 older than Context 3).

In principle, *CS.py* utilises 4 bytes of RAM per pixel of the pre-classified input image for the calculation of the feature vectors (Step 5), 1 byte per pixel for the calculation of the spatial-contextual criteria and 1 byte per pixel for the supervised post-classification (Step 6). For instance, if an input image is 10 × 10 pixels, the whole process with employ 600 bytes in the RAM. *CS.py* has been successfully tested with input files of different sizes, not having an internal limit on the size of the image to be processed (even though for large files the processing time may have a longer duration).

*CS.py* is an easy-to-use, practical and accessible plugin, downloadable as a zip package to install within QGIS, joining a family of tools for supervised object-based classification (e.g. OTB, GRASS) and providing, additionally, the possibility to include contextual information as spatial criteria to train the classification routine. As such, *CS.py* expands the potentialities of geospatial analysis to extract additional dimensions of information from remote sensing data.

## Method validation

To measure the degree of success of the post-classification results given by *CS.py*, we performed a comparison with official land use information available for the study area, choosing as dataset the “Carta degli Habitat 1:10.000” (Habitat Map 1:10.000), year 2011, created by the Sicilian Region- Assessorato Regionale Territorio e Ambiente, Dipartimento di Urbanistica, Area 2 Interdipartimentale [21]. Carta degli Habitat includes both CORINE Land Cover 2011 and Corine Biotopes (CB) [22,23] datasets at levels 4th and 5th of the classification nomenclature, thus providing combined information on both land use and land cover local features. Carta degli Habitat is also the temporally closest dataset available to our input image. However, since there is still a time discrepancy of two years between the 2013 input image and Carta degli Habitat (2011) used for the validation, expectations of the correspondence need to be somewhat scaled down due to possible changes happening in the uncovered timeframe (as already noted in [20]).

The correspondence analysis was done in QGIS using the Overlap Analysis algorithm. We calculated the correspondence of our classified conceptual spaces with CLC and CB datasets, re-adapting the correspondence method applied in Nielsen and Ahlqvist [20] in terms of mean percentage cover and Standard error of the mean (SEM) per each conceptual space VS each CLC & CB class and vice versa. We calculated the mean percentage cover and the SEM among the three conceptual spaces as well, to evaluate their overlapping dynamics, in a re-adaptation to our case of the overlapping analysis done by Ahlqvist [24] for heterogeneous land cover classes.

The results of the correspondence analysis done to measure the degree of congruence between what classified with *CS.py* as different contextual spaces of olive land use and the CLC & CB classes confirm our assumptions.

Table 2 shows the correspondence of each *CS.py* conceptual space, in terms of mean percentage cover, with the official land use dataset (both CLC & CB classes). The table shows the decreasing percentage of all the pixels in a specific *CS.py* conceptual space that overlap with the different CLC & CB classes.

**Table 2:** Correspondence (mean percentage cover) between *CS.py* conceptual spaces with both Corine Land Cover and Corine Biotopes classes.

The first CLC & CB class of correspondence for the three conceptual spaces of land use extracted with *CS.py* is the class 223/83.122 “Olive groves”/”Intensive olive groves” (mean 44.88 %).

The following four classes of correspondence (decreasing percentage) are always related to agricultural activities in the area. They are, respectively, class 21,121/82.3 “Non-irrigated arable lands”/”Extensive cultivations” (11.71 %), class 242/82.3A “Complex cultivation patters/Complex agricultural systems” (7.23 %), class 3211/34.36–34.6–34.633–34.5137–44.81 “Mediterranean and sub-Mediterranean evergreen sclerophyllous scrub” (6.17 %), and class 2311/34.81 “Meadows/Mediterranean subnitrophilous grass communities” (4.68 %). This proves the presence of olive trees, in their three different conceptual spaces, within the typical agricultural classes of this Mediterranean environment. If we sum the correspondence of all these classes, we then reach a total correspondence of 74.67 %.

The CLC & CB correspondence with the three *CS.py* conceptual spaces, in terms of mean percentage cover, is reported in Table 3. This table shows, per each CLC & CB class, the decreasing percentage of their pixels that overlap with the pixels of each of the three *CS.py* conceptual spaces.

**Table 3:** CLC & CB classes correspondence with the three *CS.py* conceptual spaces, in terms of mean percentage cover.

		Conceptual spaces of land use (%)		
Corine Land Cover	Corine Biotopes	Conceptual space 1 Orchard of century-old olive trees	Conceptual space 2 Mediterranean garden	Conceptual space 3 Modern olive plantation
**223** Olive groves	**83.112** Intensive olive groves	41.13	17.89	3.43
**31,122** Deciduous woodland	**41.732** Quereus pubeseens woods	27.06	32.04	0.68
**1112** Discontinuous urban fabric	**86.12** Discontinuous urban fabric	33.11	22.1	2.95
**2243** Eucalyptus plantations	**83.322** Eucalyptus plantations	29.13	26.16	1.24
**1122** Abandoned rural houses	**86.22** Abandoned rural buildings	30.93	18.27	5.35
**3116** Riparian woodlands	**44.81** Nerium oleander, Vitex agnus-castus, etc.	24.86	20.62	0.99
**142** Sport, leisure facilities	**85.5** Sport, leisure spaces	22.22	19.9	2.88
**143** Graveyards	**85.6** Graveyards	19.3	12.07	6.08
**3125** Conifers forests	**83.31** Conifers plantations	16.91	19.97	0.22
**242** Complex cultivation patterns	**82.3A** Complex agricultural systems	23.18	10.07	2.31
**32,222** Prunetalia	**31.81** Prunetalia **31.8A** Sub-Mediterranean deciduous thickets	20.14	7.75	0.7
**4121** Inland marshes	**53.11** Common Reed Beds (Phragmitetum)	16.43	5.2	1.05
**1111** Continuous urban fabric	**86.11** Continuous urban fabric	10.93	7.63	1.47
**1222** Road network	**86.43** Railroad and other open spaces	11.65	6.93	0.52
**2242** Broadleaved and fruit plantations	**83.325** Other broad-leaved tree plantations	10.56	8.02	0
**3211** Mediterranean and sub-Mediterranean evergreen sclerophyllous scrub	**34.36** Dry perennial grasslands **34.6** Mediterranean tall-grass steppes **34.633** Diss steppes **34.5137** Dauco-Catananchion luteae p. formations **44.81** Oleander, chaste tree, etc.	11.86	5.1	1.3
**2311** Meadows	**34.81** Mediterranean subnitrophilous grass	9.59	4.1	0.77
**221** Vineyards	**83.212** Intensive vineyards	6.38	0.53	6.38
**21,121** Non-irrigated arable lands	**82.3** Extensive cultivation	8.44	4.15	0.41

**Fig. 14:**
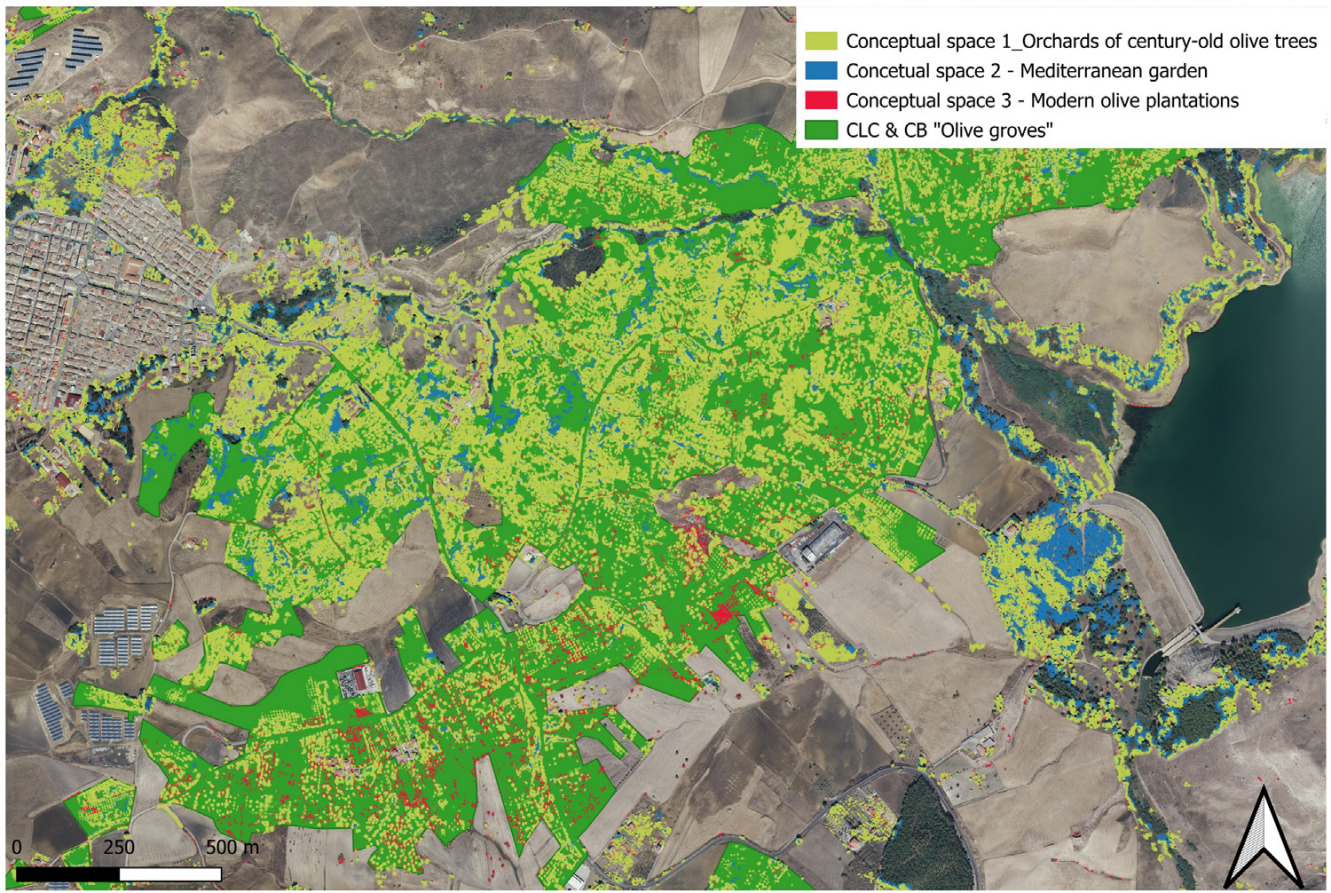
Disentanglements of the CLC & CB 223/83.122 “Olive groves” (green) into diverse conceptual land uses of the same land cover (Conceptual space 1 – Orchards of century-old olive trees in yellow, conceptual space 2 – Mediterranean garden in blue, conceptual space 3 – Modern olive plantations in red).

It is again the CLC & CB class 223/83.122 “Olive groves/Intensive olive groves” that has the higher correspondence with the three conceptual spaces (mean 20.81 % of the three conceptual spaces), with a correspondence of 41.13 % for conceptual space 1 “Orchard of century-old olive trees”, a correspondence of 17.89 % with conceptual space 2 “Mediterranean garden” and a correspondence of 3.43 % with conceptual space 3 “Intensive modern olive plantation”. These results prove that thanks to the post-classification done with *CS.py*, it is possible to disentangle a general land cover/land use class as the one 223/83.122 “Olive groves” into its many diverse conceptual spaces of land use (Fig. 14).

The remaining CLC & CB classes per decreasing correspondence are a combination of forest classes and rural areas/open spaces. Indicative the high overlap of the CLC & CB class 1122/86.22 “Abandoned rural houses” with both conceptual space 1 (30.93 %) and conceptual space 2 (18.27 %). These abandoned rural houses are *ruderi*, remnants of old rural buildings inhabited in the past by peasants. Even though this class is related to housing infrastructures, these buildings are remnants in space of a past characterised by an intense human presence in those plots for agricultural purposes. It thus makes sense the high presence of century-old olive trees (conceptual space 1) and/or other fruit trees still present in the landscape and today embedded in the garden model (e.g. pomegranate, almond, citrus trees etc.; conceptual space 2).

After the CLC & CB class 223/83.122 “Olive groves/Intensive olive groves”, the second agricultural class with a higher percentage of correspondence with the *CS.py* conceptual spaces is class 242/82.3A “Complex cultivation patterns/Complex agricultural systems (11.85 % mean), with a correspondence of 23.18 % for conceptual space 1, 10.07 % for conceptual space 2 and 2.31 % for conceptual space 3. This CLC & CB class is described as a “Mosaic of small cultivated land parcels with different cultivation types -annual crops, pasture and/or permanent crops-, eventually with scattered houses or gardens” (CLC 2011^2^). The presence and detection in this CLC & CB class of conceptual spaces centred on different spatial arrangements and uses of the olive tree shows how, with *CS.py*, it is also possible to find semantic correlations between land use classes apparently diverse (as it is the case, in this example, of CLC & CB “Olive grove” and “Complex cultivation patterns” classes). Very interestingly, we have a significant overlap of the CLC & CB “143/85.6” category “graveyard”, above all with the conceptual space 1 (19.3 %). This is due to the fact that orchards made of old-century olive trees are just located outside of the town´s graveyard space. Another interesting inference that can be made from these results is related to CLC & CB class 41.21/53.11 “Inland marshes”, which has an overlapping percentage with the conceptual space of 16.43%. Such a figure does not come as a surprise, showing instead the relatively more recent temporality of this type of watery ecosystem (naturally formed only after the construction of the artificial lake in the 70 s), compared to the location of old olive trees in the area, present in the area before the construction of the dam. The CLC & CB class 21,121/82. 3 “Non-irrigated arable land/Extensive cultivation” overlaps for 8.44 with conceptual space 1 and this makes sense since, according to the local practice, old-century olive trees are always preserved (if already present) also in arable fields.

## Limitations and concluding remarks

From the analysis done in the study area and its validation, we can conclude that the supervised contextual post-classification method presented in this paper enables the user to extract and typify from a single image of the Earth different configurations of land use characterised by the same land cover. Consequently, we can claim that *CS.py* allows the extraction of additional dimensions of information from the image data, opening new ways to approach this type of source material and generate knowledge that can be used for a better understanding of the complexity of heterogeneous landscapes. However, the analysis presented here is the result of *CS.py* implementation with only one training area per single conceptual space. More tests need to be done with the use of multiple training areas per single conceptual space, to further improve the level of details of the information extracted from one single image product. Last but not least, our work needs to be adequately accompanied by parallel research on how to develop approaches to formally represent the conceptual spaces´ semantics that emerge from this type of contextual classification (sensu [24] for instance, with the use of semantic similarity metrics as a way to overcome class heterogeneity). For such reasons, we believe further research shall be pursued on the application of *CS.py* in the many disciplinary domains investigating landscape dynamics, from quantitative and qualitative perspectives, as well as in multiple geographical environments characterised by diverse social and ecological histories.

## Ethics statements

No ethical considerations were required.

## Supplementary material *and/or* additional information

• The original data used for validation is available as Carta degli Habitat 2011 from https://www.sitr.regione.sicilia.it/download/tematismi/progetto-carta-habitat-10000/ (accessed 28 March 2024).
• The dataset created for the analysis done in this paper with *CS.py* (pre-classified input image and the three conceptual spaces/training areas created) is available from: 10.5281/zenodo.10629220.
• **Additional information**: *CS.py* is available from: 10.5281/zenodo.10615018.

License: CC BY-NC 4.0

## CRediT authorship contribution statement

**Vincenza Ferrara:** Conceptualization, Formal analysis, Data curation, Validation, Writing – original draft. **Johan Lindberg:** Software, Formal analysis. **Anders Wästfelt:** Conceptualization, Methodology, Formal analysis, Supervision, Writing – review & editing.

## Declaration of competing interest

The authors declare that they have no known competing financial interests or personal relationships that could have appeared to influence the work reported in this paper.

## Data Availability

Links to my data and code are specified in the manuscript.

## Footnotes

2 https://land.copernicus.eu/content/corine-land-cover-nomenclature-guidelines/html/index-clc-242.html.
